# Hyaluronic Acid Nanocapsules as a Platform for Needle-Free Vaccination

**DOI:** 10.3390/pharmaceutics11050246

**Published:** 2019-05-26

**Authors:** Juan I. Bussio, Carla Molina-Perea, José Vicente González-Aramundiz

**Affiliations:** 1 Departamento de Farmacia, Facultad de Química y de Farmacia, Pontificia Universidad Católica de Chile, Santiago 7820436, Chile; jibussio@uc.cl (J.I.B.); cfmolina1@uc.cl (C.M.-P.); 2 Centro de Investigación en Nanotecnología y Materiales Avanzados “CIEN-UC”, Pontificia Universidad Católica de Chile, Santiago 7820436, Chile; 3 Department of Chemical and Biomolecular Engineering, University of Notre Dame, 340E McCourtney Hall, Notre Dame, IN 46556, USA

**Keywords:** nanomedicine, vaccine, nanovaccine transcutaneous immunization, nanocapsules, hyaluronic acid, needle-free, antigen delivery, skin delivery

## Abstract

Vaccination faces many challenges nowadays, and among them the use of adjuvant molecules and needle-free administration are some of the most demanding. The combination of transcutaneous vaccination and nanomedicine through a rationally designed new-formulation could be the solution to this problem. This study focuses on this rational design. For this purpose, new hyaluronic acid nanocapsules (HA-NCs) have been developed. This new formulation has an oily nucleus with immunoadjuvant properties (due to α tocopherol) and a shell made of hyaluronic acid (HA) and decorated with ovalbumin (OVA) as the model antigen. The resulting nanocapsules are smaller than 100 nm, have a negative superficial charge and have a population that is homogeneously distributed. The systems show high colloidal stability in storage and physiological conditions and high OVA association without losing their integrity. The elevated interaction of the novel formulation with the immune system was demonstrated through complement activation and macrophage viability studies. Ex vivo studies using a pig skin model show the ability of these novel nanocapsules to penetrate and retain OVA in higher quantities in skin when compared to this antigen in the control solution. Due to these findings, HA-NCs are an interesting platform for needle-free vaccination.

## 1. Introduction

Nowadays, many immuno-preventable diseases that have re-emerged have the possibility to cause serious public health problems worldwide [1]. Due to this it is necessary now more than ever to emphasize the importance that vaccines have had on modern day health. Ever since Edward Jenner first inoculated people against smallpox in the 18th century, to the present day where vaccines are used to prevent everything from infectious diseases, certain cancers, immune-mediated disease and drug addictions, vaccination has been the most cost-effective human intervention in global health in history [2].

In spite of the great achievements made by vaccination, this area of medicine faces great challenges today. One of them has been the incorporation of new antigens such as subunit antigens, DNA, RNA, etc. Although these are safer molecules than conventional antigens; inactivated, subunit, or recombinant vaccines are generally poorly immunogenic and the use of adjuvants with them is usually necessary [3]. Another challenge that the vaccination field faces is finding new strategies in order to improve compliance, safety and logistic constraints in vaccine administration [4]. It is in this regard that the use of needles presents several problems such as the risk of injuries and infections from the reuse of needles and syringes to low patient compliance due to pain and fear of needles during immunization [5].

Nanomedicine combines pharmaceutical and biomedical sciences by making use of nanotechnology and this field is capable of giving solutions to the great challenges facing immunization [6]. The use of nanostructures as adjuvants in vaccination dates from the 1970s and throughout the years, several studies have been carried out to understand the importance of particle size in vaccination [7]. Regarding nanometric size, smaller particles have shown the highest degree of penetration around the paracortex region, have had enhanced access to dendritic cells in the lymph nodes and have generated stronger Th1 and Th2 immune responses compared to particles of a bigger size [8,9].

One of the systems of nanometric size studied for vaccination has been polymeric nanocapsules. This kind of system has the advantage of having dimensions similar to most pathogens, it can be efficiently processed by the immune system, and therefore can lead to a potent immune response [10,11]. Several raw materials have been studied as coronas in the polymeric nanocapsules developed, including proteins, polypeptides, polyaminoacids and others which have shown the capacity to deliver and control the release of encapsulated molecules, as well as increasing formulation stability in physiological conditions [12]. Moreover, this elegant core-shell structure allows the incorporation of adjuvant molecules in its oily nucleus and a polymeric shell that can transport antigens or hydrophilic molecules that could have the capacity to be easily recognized by the immune system [13] and that could also overcome biological barriers such as the skin. 

The skin is the most extensive organ in the human body and its complicated physiology makes it a physiological barrier against invasion by foreign substances and organisms. Among all the layers that the skin has, the stratum corneum acts as the major barrier as it consists of densely packed lipid mortar and protein bricks which limit active molecule permeability [14]. Additionally, the skin is a pro-inflammatory organ and immunological barrier. This is because it has immunocompetent and antigen presenting cells such as Langerhans cells, keratinocytes, dermal dendritic cells, macrophages, mast cells and T cells in its composition [15]. Moreover, skin-associated lymphoid tissue (SALT) can connect this organ with the rest of the body through a network of blood vessels and drain lymph nodes and can thus activate the immune system [16]. The high presence of immune cells in the skin allows the use of a lower amount of antigen for immune system activation and the high acceptability of transdermal administration due to various factors including; the capacity to be self-administrated, painless administration, and long-acting duration of action [17]; make the skin an attractive site for immunization.

Transdermal immunomodulation has been studied as a possibly favorable approach to treat various conditions including cancer, infectious diseases, allergies, and autoimmune diseases, among others [16]. To do this, technologies such as iontophoresis, electroporation, sonophoresis, laser ablation and microneedles (MN) have been investigated [18]. Among these techniques, MN are at the cutting edge of current investigations and they are currently the most promising. However, these technologies still have the risk of causing skin tissue and deep tissue disruption. To solve these problems, hyaluronic acid (HA) has been widely investigated as a transdermal drug delivery carrier. The rational election of this polymer is because it is one of the five major classes of macromolecules that form the extracellular matrix, promote keratinocyte proliferation, elasticity regeneration, improve wound healing, and increase the presence of retinoic acid that boosts skin hydration [19]. HA has been used in the development of polymeric nanocapsules in interesting anticancer therapy studies with excellent results [20,21]. However, for antigen delivery, hyaluronic acid nanocapsules (HA-NCs) have not been explored yet and moreover, the size of systems mentioned above are over 100 nm. This fact makes these systems unattractive for antigen delivery and it is necessary to optimize them before they can be used in antigen delivery and needle-free immunization through the skin.

Based on these facts, the aim of this study was the development of novel HA-NCs with physicochemical properties suitable for skin antigen delivery, in a special way, and that have the smallest possible size. Briefly, this novel nanosystem was characterized in order to document its physicochemical characteristics, shape, stability, ovalbumin association as a model antigen, and interaction with the immune system. Finally, ex vivo studies show the possibility of using this new nanosystem in transdermal antigen delivery and as platform for needle-free vaccination.

## 2. Materials and Methods 

### 2.1. Chemicals

Emulphor FAS-30 (EmFas-30) and benzalkonium chloride (BKC) were purchased from BASF (Ludwigshafen, Germany) and Winkler (Santiago, Chile) respectively. Lipocol HCO-40 (LP-HCO) and α tocopherol (TCPH) were donated by Vantage (Chicago, IL, USA), Hyaluronic acid (HA, 162 KDa) was provided by Bioiberica (Barcelona, Spain). Ovalbumin (OVA) was purchased from Invivogen (San Diego, CA, USA). The organic solvents HPLC grade (acetone, ethanol, acetonitrile, and methanol), Tris(hydroxymethyl)aminomethane (Tris), glycine, tween 20 and glycerol were obtained from Merck (Darmstadt, Germany). Tetramethylethylenediamine (TEMED), Bromophenol blue and ammonium persulfate (APS) were purchased from Amresco (Solon, OH, USA). Thirty per cent Acrylamide/Bis solution, Goat Anti-mouse IgG horseradish peroxidase conjugate, Goat Anti-Rabbit IgG horseradish peroxidase conjugate and Clarity™ Western ECL Substrate, Precision Plus Protein™ All Blue were supplied by Bio-rad (Hercules, CA, USA). MEM eagle solution, Roswell Park Memorial Institute medium (RPMI), fetal bovine serum (FBS), Penicillin-streptomycin solution and DPBS were obtained from Biological Industries (Cromwell, CT, USA). Rabbit polyclonal antibody Anti-OVA and Mouse monoclonal antibody Anti-C3/C3b were supplied by Rockland Inc (Lymeric, PA, USA) and Abcam (Cambrige, UK) respectively. AlamarBlue and 3,3′,5,5′-Tetramethylbenzidine (TMB) were obtained from Sigma Aldrich (Darmstadt, Germany), finally Cobra Venom Factor was purchased from Quidel (San Diego, CA, USA) and Buffer Veronal was obtained from Lonza (Basel, Switserland).

### 2.2. Preparation and Characterization of Nanosystems

Nanosystems were designed and developed based on a previous experimental design made by our research group using the solvent displacement technique [13,22]. The organic phase consisted of 10 mg of BKC, 60 or 120 mg of TCPH and 50 or 100 mg of LP-HCO plus organic solvents (750 µL ethanol and 4.25 mL of acetone). The aqueous phase consisted of 10 mL of either water for nanoemulsion preparation or HA solution 0.5 mg/mL for one-step hyaluronic acid nanocapsules preparation (HA-NCs). The organic solvents were eliminated by evaporation under vacuum (Rotavapor Heidolph, Germany) to a constant volume of 5 mL. For the two-step HA-NCs preparation method, we incubated the primary NE with HA (2 mg/mL). The volume ratio used was 1:1 (NE:HA solution), the final theoretical concentration of HA was 1 mg/mL for both nanocapsule formulations.

The isolation process of the formulations was made through centrifugation (Universal 320R, Hettich, Germany) at 35,200 RCF for 2 h at 4 °C. The nanosystems were then resuspended in the same amount of ultrapure water that was used initially.

Mean particle size and the polydispersity index were determined by Photon correlation spectroscopy, samples were diluted with ultrapure water and the measurement was carried out at 25 °C. Superficial charge was measured by diluting the samples with 1 mM KCl and then quantified by laser-Doppler anemometry. Both analyses were performed using Zetasizer^®^, NanoZS (Malvern Instruments, Malvern, UK). Scanning Transmission Electron Microscopy (STEM) (Inspect 50, Holland) was carried out to determine the morphology of the nanosystems. For this purpose, the nanosystems were deposited on carbon coated copper grids and were stained with phosphotungstic acid solution 2% (*w*/*v*).

### 2.3. Colloidal Stability in Storage and Physiological Conditions

The stability of HA-NCs in storage condition was carried out by analysing the mean particle size and zeta potential evolution for 24 months. The aqueous suspensions were stored at 4 °C. In order to study stability in physiological conditions or in cell culture medium (which was done for future cell viability studies), HA-NCs were incubated in PBS or supplemented RPMI at 37 °C (pH = 7.4) and formulation stability was evaluated by motoring the evolution of particle size for 48 h. 

### 2.4. Ovalbumin Association to the Hyaluronic Acid Nanocapsules 

HA-NCs were incubated in equal volumes with Ovalbumin (OVA) in 2:1 HA:antigen mass ratios (theoretical amount of HA adsorbed on the nanocapsules’ surface). For example, for 1 mL of OVA loaded HA-NCs, 0.5 mL of HA-NCs was incubated with 0.5 mL of OVA 0.5 mg/mL, thus obtaining an OVA concentration of 0.25 mg/mL adsorbed in the nanocapsules. The process was begun by isolating the blank formulation (Section 2.2) and then incubating the isolated formulation with OVA solution at room temperature (RT) for 1 h. The OVA-loaded HA-NCs were isolated and the physicochemical properties of the nanosystems were analysed as previously described (Section 2.2). HA-NCs capacity to associate OVA as the model antigen was quantified by ELISA. Isolated HA-NCs loaded with OVA were treated with acetonitrile (AcN) in a ratio of 4:5 (AcN:NC) and 100 µL of each of the samples was incubated overnight at 4 °C in an EIA/RIA 96-well plate (Corning, Corning, NY, USA). After several washes the plates were blocked with 5% PBS-non-fat milk for 2 h at RT. Rabbit anti-OVA Ab was diluted (1:10,000) and incubated for 2 h at RT. Goat anti-rabbit IgG (secondary Ab) was added (diluted to 1:3000) and incubated for 2 h at RT. Intensive washes were performed between all of these procedures. The wells were revealed by adding 3,3′,5,5′-Tetramethylbenzidine. After 20 min, the reaction was stopped with 50 µL of H_2_SO_4_ 2N. The absorbance was measured at 450 nm (Stat Fax 2100, Awareness Technology, Ramsey, MN, USA).

### 2.5. Cell Viability Studies

Alamar blue was used to analyze the effect of HA-NCs and its nanoemulsion on cell viability. 1 × 10^5^ Raw 246.7 cells/well (ATCC, Manassas, VA, USA) were cultured in RPMI supplemented with 5% fetal bovine serum (FBS), 1% (*v*/*v*) MEM eagle solution, and 1% (*v*/*v*) penicillin-streptomycin at 37 °C in a humidified atmosphere containing 5% carbon dioxide. The RAW 264.7 cell line was obtained from Dr. Mario Faúndez C. Eight different concentrations (25–0.195 mg/mL) of isolated HA-NCs or NE were incubated with the cells. Triton X-100 (1:10 dilution in RPMI) was used as the positive control and untreated cells as the negative control. After 4 h of incubation, the treatments were removed and replaced with fresh RPMI. At 24 h, the medium was eliminated and Alamar Blue diluted in RPMI was added. Cell viability was quantified according to the manufacturer`s instructions.

### 2.6. Activation of Complement Cascade Studies 

The study of complement cascade activation induced in vitro by HA-NCs was performed by analysing the C3/C3b cleavage products using Western blot technique [23]. To this effect, a pool of human plasma from healthy donors was incubated with equal volumes of veronal buffer and different concentrations of HA-NCs (2–0.5 mg/mL). Cobra venom factor (CVF) and dPBS were used as positive or negative controls, respectively. The formulations and controls were mixed and incubated at 37 °C for 30 min. Five microliters of each sample was loaded on a 10% SDS-PAGE gel and subjected to electrophoresis and transferred to a PVDF membrane (Immun-Blot, Biorad, Hercules, CA, USA). Membranes were blocked for 1 h at RT (non-fat milk 5% (*w*/*v*)) and subsequently they were incubated overnight with mouse mAb against human C3/C3b (1:2000) at 4 °C. After intensive washes, the membranes were incubated with goat anti-mouse IgG (secondary antibodies (1:3000)) for 1 h at RT. Finally, the membranes were revealed using Clarity™ Western ECL Substrate (Chemidoc, Bio-Rad, Hercules, CA, USA). To quantify the C3b cleavage, the intensity of bands was normalized to the positive control (giving it a value of 1) using ImageLab (Bio-rad, Hercules, CA, USA). 

### 2.7. Skin Permeation Study

Pig skin (from 2–3 day old pigs) was used as the model to predict the permeation of OVA from HA-NCs [24]. Different regions (hind leg, fore leg, back) of the skin were selected and any hair was peeled before each experiment. The diffusion area was of 4.15 cm^2^ using Franz vertical diffusion cells Laboratory Glass Apparatus Inc., Berkeley, CA, USA) and the temperature (37 °C) and agitation (300 rpm) remained constant throughout the experiment. The donor solution was 1 mL of OVA loaded HA-NCs or OVA in PBS (0.25 mg/mL). The amount of OVA was 250 µg for each experiment. The receptor solution was PBS. At 24 h, the receptor solution was placed in an oven (Fisher Isotemp^®^ Oven Senior Model, Waltham, MA, USA) at 50 °C until its complete evaporation. The amount of OVA that was retained in the skin was also quantified. At the end of the experiment, the skin was washed with PBS and deposited in 15 mL of methanol for 16 h. Then, the skin was subsequently discarded and the samples were treated using the same method that was used for the receptor solution.

Samples with evaporated solvent were reconstituted in 1 mL of ultrapure water and analysed by quantitative Western blot [25]. Briefly, samples were reduced with β-mercaptoethanol and loaded onto an acrylamide gel. As a control, different concentrations of OVA (50–2 ng) were reduced and loaded onto the same gel. Gel and membranes were treated as described in Section 2.7. The only difference between these procedures were the antibodies used. The primary Ab was rabbit polyclonal antibody Anti-OVA and the secondary Ab was Goat Anti-Rabbit IgG. 

### 2.8. Data Analysis 

Results are represented as Mean ± SD of at least three measurements (six in the case of skin permeation study). Statistical studies (complement activation and transdermal experiments) were both analysed by *t*-student. Differences were considered significant at *p* < 0.05. 

### 2.9. Ethical Issues

All Protocols and experiments developed in this work were approved by the Ethical and Biosecurity Committee of Facultad de Química, Pontificia Universidad Católica de Chile for the project “Fondecyt de Inicio 11140797” with date October 24th, 2014.

## 3. Results and Discussion

The main goal of this work was to develop novel hyaluronic acid nanocapsules (HA-NCs) that can overcome the stratum corneum and interact with the immune system in order to achieve antigen transdermal delivery. Hyaluronic acid (HA) was chosen as a corona for various reasons: its biosafety, biocompatibility, and because of its use as a material in biomedical applications [26]; its low molecular weight fragmentation products could activate immune cells through activation or regulation of Toll-like receptors 4 and 2 and by stimulating the secretion of different cytokines [27,28]; it is a product that has been frequently used in the cosmetic and nutricosmetic industry [29]. The use of polymeric nanocapsules as a vehicle in transdermal antigen delivery is due to their structure (nucleus-corona) which allows the incorporation of oils and hydrophobic molecules with immunoadjuvant properties in the nucleus and of hydrophilic active molecules (such as an antigen or a drug) that are embedded in the polymeric shell [30].

### 3.1. Hyaluronic Acid Nanocapsules Preparation and Characterization

**Figure 1:**
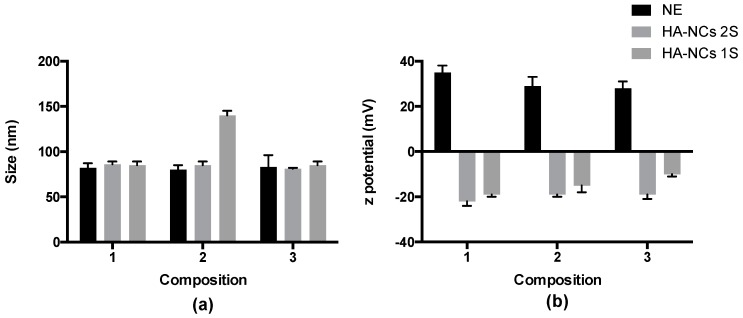
Physicochemical characterization of nanosystems according to their hydrodynamic diameter (**a**) and superficial charge (**b**). Composition 1: 50 mg LP-HCO and 60 mg TCPH; composition 2: 50 mg LP-HCO and 120 mg TCPH and composition 3: 100 mg LP-HCO and 60 mg TCPH. The amount of benzalkonium chloride was always constant (10 mg). NE: nanoemulsion, HA-NCs: hyaluronic acid nanocapsules (2S: two steps, 1S: 1 step). TCPH: α tocopherol and LP-HCO: Lipocol HCO-40.

Recently, our research group has published a work about chitosan nanocapsules that are smaller in size and their use in antigen transdermal delivery [31]; sizes under 100 nm facilitate the absorption through the skin. In particle vaccine delivery, this small size influences lymph node trafficking and localization, cellular distribution within the lymph nodes, and also increases efficiency of cross-presentation [32]. Following this rational design, HA-NCs were prepared by solvent displacement technique. TCPH was chosen due to its immune system stimulating properties [33] and an adequate combination of LP-HCO and BKC was necessary in order to stabilize the system. To find the formulation with the smallest size and the superficial charge that ensured the best possible stability, TCPH and LP-HCO masses were optimized by maintaining the BCK concentration constant. The physicochemical characterizations of the different nanosystems are shown below in Figure 1. 

The nanosystems designed and prepared were: nanoemulsions (all components without HA); HA-NCs prepared in two steps (a nanoemulsion incubated with HA solution); and HA-NCs prepared in one step (the most attractive method because of its capacity to be scaled to an industrial level). All formulations had a size smaller than 100 nm except the HA-NCs prepared in one step with double TCPH (120 mg). By having more oil in the formulation, the size of polymeric nanocapsules was bigger. This is in accordance with literature which states that when there is less oil in the organic phase, the size of polymeric nanocapsules is smaller [34]. The polydispersity index (PDI) was always lower than 0.25 for all formulations, indicating that the formulations are homogeneous, and they have only one particle population. The three nanoemulsions (NE) had a high positive zeta potential owing to presence of BKC in the emulsion. This cationic surfactant was used to favour electrostatic interaction between the oil drops and the negative charge of HA. Also, this polymer decreased the superficial charge of all HA-NCs formulations that had a negative zeta potential and this property was independent of the preparation method used. This negative zeta potential value indicated the presence of HA in the corona of the nanocapsules. The formulation that had a lower size and that used a smaller number of excipients for preparation was HA-NCs composition one, prepared in one step; for this reason, this nanosystem was considered for the following experiments.

**Figure 2:**
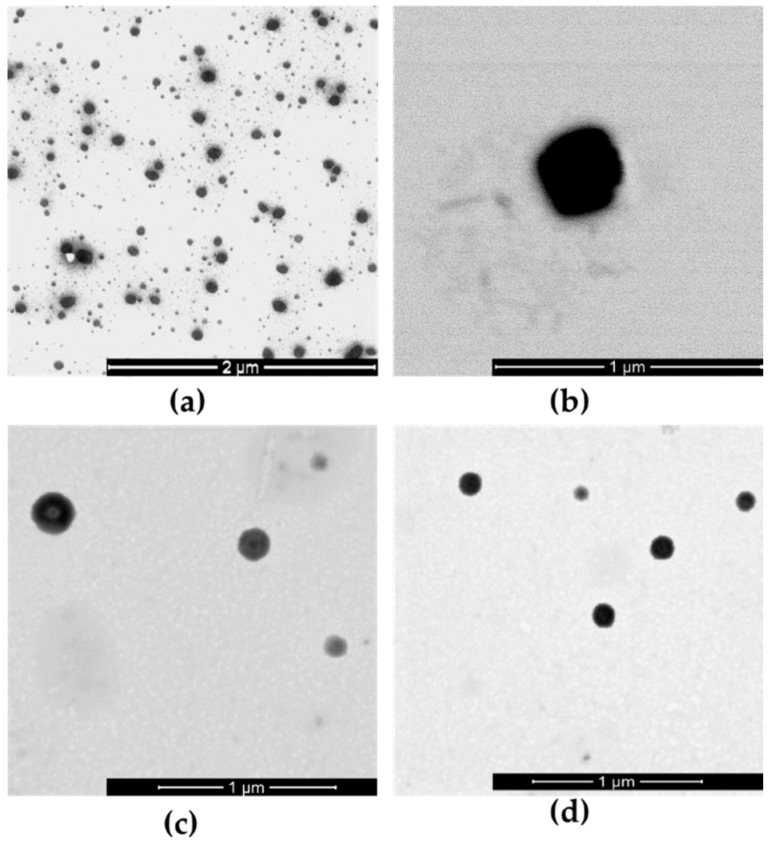
Scanning Transmission Electron Microscopy (STEM) images of hyaluronic acid nanocapsules (HA-NCs). (**a**,**b**): Formulation without antigen; (**c**,**d**): ovalbumin-loaded HA-NCs.

The shape and morphology of HA-NCs was evaluated by Scanning Transmission Electron Microscopy (STEM). Figure 2 shows STEM images where it is possible to see HA-NCs have spherical shape and a homogenous population.

### 3.2. Colloidal Stability in Storage and Physiological Conditions

**Figure 3:**
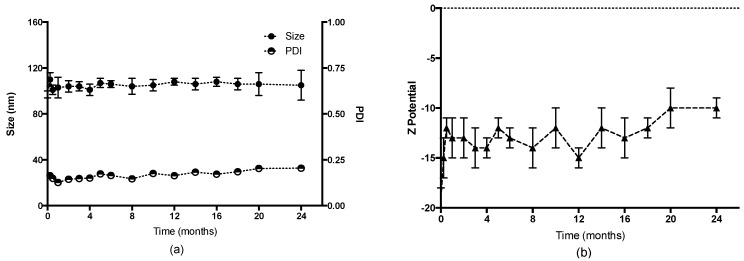
Stability of Hyaluronic acid nanocapsules at 4 °C for 24 months. (**a**) Particle size and polydispersity index (PDI), (**b**) zeta potential.

In order to evaluate the stability of HA-NCs in storage conditions, this formulation was kept at 4 °C and the evolution of physicochemical characteristics was evaluated. As Figure 3 shows, HA-NCs maintained their particle size (~100 nm) and their PDI lower than 0.25 for 2 years. The superficial charge remained negative which indicates the presence of HA as a shell of this formulation. These results indicate that this formulation has a homogeneous particle population for at least 24 months. The amount of HA present in the shell allows the repulsion between the formulation particles and this is evidenced through the high stability in aqueous suspension.

**Figure 4:**
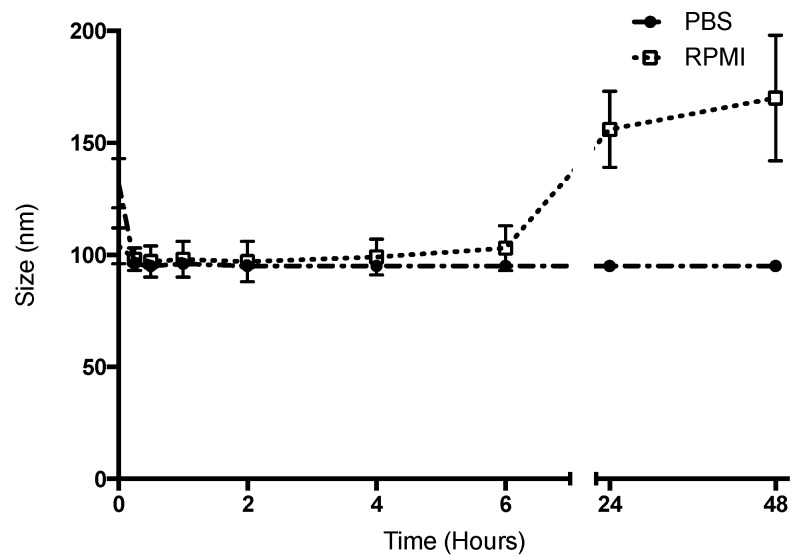
Stability of Hyaluronic acid nanocapsules at 37 °C for 48 h in physiological conditions (PBS, black circles) and cell culture media buffer (supplemented RPMI, white squares).

The stability of the HA-NCs in simulated physiological conditions was assessed by measuring the changes in size of this formulation through time at a high temperature (37 °C) in PBS or cell culture media buffer (RPMI, pH: 7.4). Particle size can reflect the presence of aggregation and system destabilization. As shown in Figure 4 after 48 h the droplets maintained their nanometric size, although there was an increase of 50 nm at 24 h (in the case of RPMI), which was not considered significant. 

The difference between PBS and RPMI at 24 and 48 h could be due to RPMI which is supplemented with FBS, MEM eagle solution and penicillin-streptomycin, and one of these could have been the cause of the slight increase in size. This size increase occurred at 24 h and we hope that the interaction between HA-NCs and antigen presenting cells occur during the first 2 h (at which point they have their original size). Moreover, the formulation at 24 h in RPMI had a polydispersity index of 0.304. This value is considered to be acceptable and indicates a homogenous population [35]. One possibility of formulation optimization to increase stability in RPMI is to increase the HA density to enhance repulsion between the particles. The possibility to increase the density of HA in the shell could be through the increase of the benzalkonium chloride concentration, because an increase of positive charge in the oily droplets could increase the HA deposit. However, this increase of the cationic surfactant could be reflected in an increase in the cytotoxicity of our formulation. Another possibility to optimize the formulation could be to change the cationic surfactant to one that has shown a lower cytotoxicity at the same concentration. Cationic surfactants such as alkyltrimethylammonium bromide, octylamine and 1-decyl-3-methylimidazolium have recently shown a lower cytotoxicity than benzalkonium chloride and its possible to design and prepare systems with nanometric size using these surfactants [36].

### 3.3. Ovalbumin Association to the Hyaluronic Acid Nanocapsules

Several ways to encapsulate ovalbumin (OVA) as a model antigen to the HA-NCs were studied. The first attempt was to include OVA in the organic phase together with the oily nucleus. There is evidence that it is possible to encapsulate proteins and peptides in the oily nucleus of polymeric nanocapsules [37]. Nevertheless, when OVA was included in the organic phase of HA-NCs, the result was aggregation of the system. This could be due to low solubility of this model antigen with the solvents used in the organic phase. Another approach was changing the protein’s pH to at least 2 units under the isoelectric point (4.5), which was done to favour positively charged molecules which were then incubated with HA-NCs. In this way, it was expected that the positive charge of OVA with the negative superficial charge of HA-NCs would help to promote association among them through electrostatic interaction. Although, this approach had good results for other antigens (such as influenza antigen [13]), the pH changes of the OVA solution and the association of HA-NCs resulted in the aggregation of the systems again. Finally, in order to associate OVA to HA-NCs, this model antigen was incubated with the isolated formulation without changing protein pH (6.9) at RT. The association of the model antigen did not change the physicochemical properties of the HA-NCs (Table 1). 

The size stayed below 100 nm with a low polydispersity index that reflects only one homogeneous family of particles. The shape of this loaded formulation was evaluated by STEM images to confirm the photon correlation spectroscopy results (Figure 2c,d). The superficial charge of OVA-loaded HA-NCs remained negative thus indicating the presence of HA as a shell. The capacity of isolated formulations to associate OVA to HA-NCs was quantified through ELISA. OVA association was 67%, where hydrophobic interactions, hydrogen bonds and Van der Waals forces predominated. This high value was similar to those obtained for this antigen associated in other similar nanosystems [38,39]. Western blot analysis was performed because this assay has diverse applications to demonstrate events such as protein turnover, protein abundance, protein-protein interactions, or modifications such as events of cleavage, phosphorylation, ubiquitinylation, glycosylation, methylation and SUMOylation [40]. None these events occurred after OVA HA-NCs adsorption and the structure and the integrity of the model antigen was not affected by HA-NC association.

### 3.4. Cell Viability

**Figure 5:**
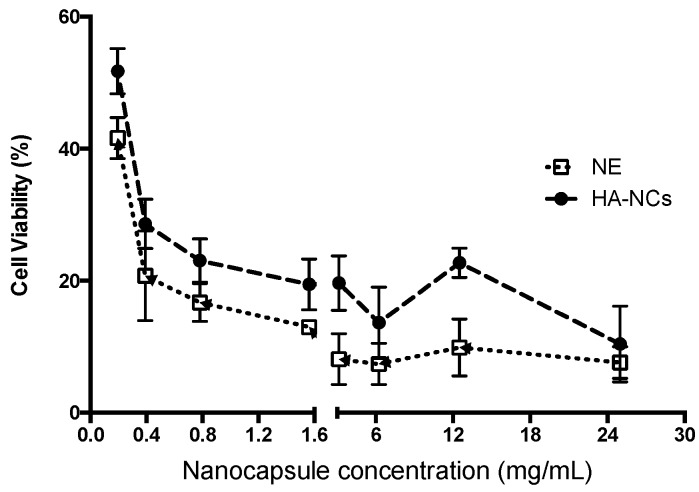
Cytotoxic effect of Hyaluronic acid nanocapsules (HA-NCs, black circles) and its nanoemulsion (NE, white square). RAW 264.7 was incubated with different concentrations of each formulation for 4 h and after 24 h was analysed by Alamar Blue.

In order to evaluate the cytotoxicity of HA-NCs and their nanoemulsion, increasing concentrations of these nanosystems were incubated with macrophages for 4 h (Figure 5). Previous studies have shown that nanosystems maintain their physicochemical properties in cell culture media buffer during this time (Section 3.2). Both formulations showed a dose-dependent trend in macrophage viability. No significant differences between formulations were observed, however nanoemulsions showed a higher cytotoxicity to nanocapsules at the same concentration. This may be due to the positive surface charge of the nanoemulsion, which may present a greater cytotoxicity. The negative charge of the cell membranes allowed positive particles to be easily ingested, thus increasing cell toxicity by electrostatic interactions [41]. The small size of these systems could play a crucial role in cell viability because bigger particle sizes or even bulk materials that do not show toxicity could increase cytotoxicity when their particle size is reduced [42]. Moreover, very small nanosystems can easily enter cells and then translocate into different organelles thus being able to induce greater cytotoxicity [43]. A wide range of nanosystem concentrations was evaluated, however, the concentrations studied were much higher than we expect to have in vivo conditions.

### 3.5. Activation of Complement Cascade Studies

Complement activity regulates inflammatory and immunity functions in the body. It is crucial in the innate immune system because it is a line of defence against different external pathogens. This system is composed of more than 50 plasma and cell surface proteins that act as a cascade and promote opsonization, phagocytosis, inflammation, recognition, and clearance of microorganisms [44]. This system can be activated through three distinct pathways: the classical, lectin, and alternative pathway but C3 degradation is a common step in all 3 of these pathways [45]. In the development of novel adjuvants for vaccine delivery, the activation of the complement cascade has been studied because of its important role in innate and adaptive immunity and their antigen-specific response [46]. Table 2 shows the results of C3 degradation induced by HA-NCs at different concentrations. This Western blot analysis was expressed as relative values to the Cobra Venom Factor (CVF) that was used as the positive control. Activation of the complement was positive when C3 degradation produced by HA-NCs had statistically significant differences with respect to the negative control (dPBS) [47]. A wide range of concentrations (2.0–1.25 mg/mL) of HA-NCs induced complement activation. These results showed that the developed HA-NCs have adjuvant properties like those shown by aluminium salts (the classical adjuvant used in modern day vaccines). It has been shown that the immune-enhancing and adjuvant effect of aluminium salts could be because these compounds activate the complement cascade, contribute to the recruitment of inflammatory cells to the site of administration, and induce the activation of inflammasome [48].

### 3.6. Skin Permeation Study

Pig skin has been widely used as a model to predict the permeation of novel drugs through human skin. This membrane has shown similar thickness, lipophilicity, and follicular and histological structure when compared to human skin [49,50]. For this ex vivo experiment: different parts of pig skin such as back, hind and fore leg were used to represent different skin conditions that human skin can have [51]; the data represents the mean of these different regions. Franz cells were used with an area of 4.15 cm^2^, and temperature and agitation remained constant. One millilitre of OVA loaded HA-NCs or OVA in PBS (0.25 mg/mL) was used as donor solution. At 24 h the receptor solution and the skin were analysed to obtain the amount of OVA that permeated and was retained in the skin, respectively.

**Figure 6:**
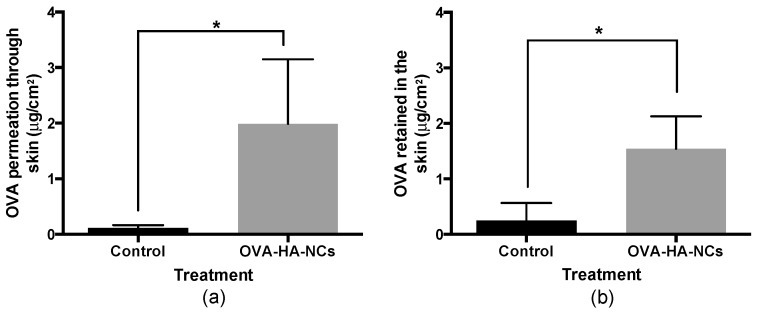
Effect of hyaluronic acid nanocapsules in the permeation (**a**) and retention (**b**) of ovalbumin (OVA) in pig skin. Control was OVA in solution (0.25 mg/mL). OVA-HA-NCs: OVA loaded hyaluronic acid nanocapsules. (*p* < 0.05).

Figure 6a shows that OVA associated to HA-NCs has a higher diffusion than control (same amount of antigen in solution). These are great results, especially when considering “the 500 Dalton rule” which states that molecules with a higher molecular weight than 500 Da cannot pass the corneal layer due to the physicochemical properties of the skin [52]. It is important to note that OVA molecular weight is 43 kDa and when its associated to HA-NCs, OVA had a penetration rate 22 times higher than OVA in solution. These results demonstrate the efficiency of HA-NCs as vehicle in the absorption of big molecules such as proteins or antigens through the skin.

The amount of OVA associated to HA-NCs that was retained in the skin was 33 times higher than OVA in solution (Figure 6b). This amount of OVA could slowly be released to the deeper stratums of the skin or interact with Langerhans cells, T cells and dermal dendritic cells to improve the immune response. Moreover, keratinocytes, the most abundant cells in the epidermis, express TLR that could be activated by the HA present in our formulation and recognize the pathogen-associated molecular patterns of invading antigens and thus trigger an efficient immune response [16].

Some studies have shown that HA may facilitate the retention and localization of drugs in the skin as well as increase drug absorption through the skin [53]. Nevertheless, the exact mechanism for transdermal transport remains unclear. The possible mechanisms of how these glycosaminoglycan coated nanocapsules are able to overcome the SC and deliver OVA inside the skin are similar to proposed mechanisms for how this polymer facilitates drug transport through the skin. First, the absorption of OVA facilitated by HA-NCs could be mediated by receptors or co-transports that are widely distributed in resident skin cells (HA receptors are highly expressed in keratinocytes in the epidermis and fibroblasts in the dermis). These structures may localize this polysaccharide and increase OVA transdermal delivery [54]. Another possibility is that the HA-NCs produce a hydration effect in the stratum corneum, it may swell and form paths for transport of OVA through the outermost stratum corneum thus facilitating dermal OVA absorption and could also aid in the retention of this antigen within the more hydrated epidermal layers [55]. Finally, in its backbone, HA present in the shell of the developed nanocapsules has hydrophobic patch domains of eight CH groups, these could make a complex with phospholipids and disrupt the stratum corneum, enhancing the skin permeability and facilitating OVA absorption through this organ [56].

## 4. Conclusions

Novel hyaluronic acid nanocapsules (HA-NCs) were designed and developed for a transdermal antigen delivery approach. This formulation had a size smaller than 100 nm and a negative superficial charge because of the presence of hyaluronic acid as a shell in the system. Their morphology was spherical and the formulation had only one particle population. This nanosystem showed high colloidal stability when stored at 4 °C (for at least two years) and in physiological conditions (at least 48 h). In addition, HA-NCs showed high capacity to associate OVA as a model antigen and activate the complement cascade thus proving its adjuvant properties. Finally, these vehicles incremented skin absorption of the model antigen at a rate 22 times higher than OVA in solution and retained the antigen in the skin 33 times more when compared to the same control. These results suggest the promising potential of HA-NCs in antigen transdermal delivery as a platform for needle free vaccination.

## Figures and Tables

**Table 1:** Physicochemical characterization of hyaluronic acid nanocapsules (HA-NCs) loaded with ovalbumin (OVA) as model antigen. PDI: polydispersity index.

Nanosystem	Size (nm)	PDI	Potential z (mv)	% OVA Association
HA-NCs blank	85 ± 4	0.175	−19 ± 1	
OVA loaded HA-NCs	95 ± 5	0.132	−22 ± 2	
OVA loaded HA-NCs (isolated)	93 ± 4	0.128	−20 ± 1	67 ± 5

**Table 2:** Complement activation of human plasma induced by different Hyaluronic acid nanocapsule (HA-NCs) concentrations. The values correspond to the intensity band of Western blot normalized to the Cobra Venom Factor (CVF, positive control). * denotes a significant difference between treatment and the negative control (dPBS). (*p* < 0.05).

Treatment	Amount/Concentration	Normalized Cleaved Protein
Positive control	10 μg CVF	1 *
Negative control	dPBS	0.35 ± 0.04
HA-NCs	2 mg/mL	0.82 ± 0.15 *
	1.75 mg/mL	0.72 ± 0.13 *
	1.25 mg/mL	0.73 ± 0.17 *
	0.75 mg/mL	0.42 ± 0.07
	0.5 mg/mL	0.45 ± 0.14

## References

[1] Siani A. (2019). Measles outbreaks in Italy: A paradigm of the re-emergence of vaccine-preventable diseases in developed countries. Prev. Med..

[2] Bragazzi N.L., Gianfredi V., Villarini M., Rosselli R., Nasr A., Hussein A., Martini M., Behzadifar M. (2018). Vaccines Meet Big Data: State-of-the-Art and Future Prospects. From the Classical 3Is (“Isolate-Inactivate-Inject”) Vaccinology 1.0 to Vaccinology 3.0, Vaccinomics, and Beyond: A Historical Overview. Front. Public Health.

[3] Bonam S.R., Partidos C.D., Halmuthur S.K.M., Muller S. (2017). An Overview of Novel Adjuvants Designed for Improving Vaccine Efficacy. Trends Pharmacol. Sci..

[4] Schulze K., Ebensen T., Riese P., Prochnow B., Lehr C.-M., Guzmán C.A., Stadler M., Dersch P. (2016). New Horizons in the Development of Novel Needle-Free Immunization Strategies to Increase Vaccination Efficacy. How to Overcome the Antibiotic Crisis: Facts, Challenges, Technologies and Future Perspectives.

[5] Singh B., Maharjan S., Sindurakar P., Cho K.H., Choi Y.J., Cho C.S. (2018). Needle-Free Immunization with Chitosan-Based Systems. Int. J. Mol. Sci..

[6] Bobo D., Robinson K.J., Islam J., Thurecht K.J., Corrie S.R. (2016). Nanoparticle-Based Medicines: A Review of FDA-Approved Materials and Clinical Trials to Date. Pharm. Res..

[7] González-Aramundiz J.V., Cordeiro A.S., Csaba N., De la Fuente M., Alonso M. (2012). Nanovaccine: Nanocarriers for antigen delivery. Biol. Aujourd’hui.

[8] Kumar S., Anselmo A.C., Banerjee A., Zakrewsky M., Mitragotri S. (2015). Shape and size-dependent immune response to antigen-carrying nanoparticles. J. Control. Release.

[9] Howard G.P., Verma G., Ke X., Thayer W.M., Hamerly T., Baxter V.K., Lee J.E., Dinglasan R.R., Mao H.-Q. (2019). Critical size limit of biodegradable nanoparticles for enhanced lymph node trafficking and paracortex penetration. Nano Res..

[10] Lou B., De Beuckelaer A., Boonstra E., Li D., De Geest B.G., De Koker S., Mastrobattista E., Hennink W.E. (2019). Modular core-shell polymeric nanoparticles mimicking viral structures for vaccination. J. Control. Release.

[11] González-Aramundiz J.V., Presas E., Dalmau-Mena I., Martínez-Pulgarín S., Alonso C., Escribano J.M., Alonso M.J., Csaba N.S. (2017). Rational design of protamine nanocapsules as antigen delivery carriers. J. Control. Release.

[12] González-Aramundiz J.V., Lozano M.V., Sousa-Herves A., Fernandez-Megia E., Csaba N. (2012). Polypeptides and polyaminoacids in drug delivery. Expert Opin. Drug Deliv..

[13] Vicente S., Peleteiro M., Gonzalez-Aramundiz J.V., Diaz-Freitas B., Martinez-Pulgarin S., Neissa J.I., Escribano J.M., Sanchez A., Gonzalez-Fernandez A., Alonso M.J. (2014). Highly versatile immunostimulating nanocapsules for specific immune potentiation. Nanomedicine.

[14] Nagula R.L., Wairkar S. (2019). Recent advances in topical delivery of flavonoids: A review. J. Control. Release.

[15] Yazdi A.S., Röcken M., Ghoreschi K. (2016). Cutaneous immunology: Basics and new concepts. Semin. Immunopathology.

[16] Zhao Z., Ukidve A., Dasgupta A., Mitragotri S. (2018). Transdermal immunomodulation: Principles, advances and perspectives. Adv. Drug Delivery. Rev..

[17] Mofidfar M., O’Farrell L., Prausnitz M.R. (2019). Pharmaceutical jewelry: Earring patch for transdermal delivery of contraceptive hormone. J. Control. Release.

[18] Lee H., Song C., Baik S., Kim D., Hyeon T., Kim D.-H. (2018). Device-assisted transdermal drug delivery. Adv. Drug Delivery. Rev..

[19] Nashchekina Y.A., Raydan M. (2018). Noninvasive penetration of 5 nm hyaluronic acid molecules across the epidermal barrier (in vitro) and its interaction with human skin cells. Skin Res. Technol..

[20] Teijeiro-Valiño C., Novoa-Carballal R., Borrajo E., Vidal A., Alonso-Nocelo M., de la Fuente Freire M., Lopez-Casas P.P., Hidalgo M., Csaba N., Alonso M.J. (2019). A multifunctional drug nanocarrier for efficient anticancer therapy. J. Control. Release.

[21] Oyarzun-Ampuero F.A., Rivera-Rodriguez G.R., Alonso M.J., Torres D. (2013). Hyaluronan nanocapsules as a new vehicle for intracellular drug delivery. Eur. J. Pharm. Sci..

[22] Bussio J., Molina-Perea C., González-Aramundiz J. (2018). Lower-Sized Chitosan Nanocapsules for Transcutaneous Antigen Delivery. Nanomaterials.

[23] Neun B.W., Dobrovolskaia M.A., McNeil S.E. (2011). Qualitative Analysis of Total Complement Activation by Nanoparticles. Characterization of Nanoparticles Intended for Drug Delivery.

[24] Abd E., Yousef S.A., Pastore M.N., Telaprolu K., Mohammed Y.H., Namjoshi S., Grice J.E., Roberts M.S. (2016). Skin models for the testing of transdermal drugs. Clin. Pharmacol. Adv. Appl..

[25] Dickinson J., Fowler S., Walker J. (2002). Quantification of Proteins on Western Blots Using ECL. The Protein Protocols Handbook.

[26] Tiwari S., Bahadur P. (2019). Modified hyaluronic acid based materials for biomedical applications. Int. J. Biol. Macromol..

[27] Fallacara A., Baldini E., Manfredini S., Vertuani S. (2018). Hyaluronic Acid in the Third Millennium. Polymers.

[28] González-Aramundiz J.V., Peleteiro Olmedo M., González-Fernández Á., Alonso Fernández M.J., Csaba N.S. (2015). Protamine-based nanoparticles as new antigen delivery systems. Eur. J. Pharm. Biopharm..

[29] Bukhari S.N.A., Roswandi N.L., Waqas M., Habib H., Hussain F., Khan S., Sohail M., Ramli N.A., Thu H.E., Hussain Z. (2018). Hyaluronic acid, a promising skin rejuvenating biomedicine: A review of recent updates and pre-clinical and clinical investigations on cosmetic and nutricosmetic effects. Int. J. Biol. Macromol..

[30] Crecente-Campo J., Alonso M.J. (2019). Engineering, on-demand manufacturing, and scaling-up of polymeric nanocapsules. Bioeng. Transl. Med..

[31] Gonzalez-Aramundiz J.V., Peleteiro Olmedo M., González-Fernández A., Alonso Fernandez M.J., Csaba N.S. (2018). Protamine Nanocapsules for the Development of Thermostable Adjuvanted Nanovaccines. Mol. Pharm..

[32] Gause K.T., Wheatley A.K., Cui J., Yan Y., Kent S.J., Caruso F. (2017). Immunological Principles Guiding the Rational Design of Particles for Vaccine Delivery. ACS Nano.

[33] Kawai M., Nakamura T., Miura N., Maeta M., Tanaka H., Ueda K., Higashi K., Moribe K., Tange K., Nakai Y. (2018). DNA-loaded nano-adjuvant formed with a vitamin E-scaffold intracellular environmentally-responsive lipid-like material for cancer immunotherapy. Nanomed. Nanotechnol. Biol. Med..

[34] Abellan-Pose R., Teijeiro-Valiño C., Santander-Ortega M.J., Borrajo E., Vidal A., Garcia-Fuentes M., Csaba N., Alonso M.J. (2016). Polyaminoacid nanocapsules for drug delivery to the lymphatic system: Effect of the particle size. Int. J. Pharm..

[35] Danaei M., Dehghankhold M., Ataei S., Hasanzadeh Davarani F., Javanmard R., Dokhani A., Khorasani S., Mozafari M.R. (2018). Impact of Particle Size and Polydispersity Index on the Clinical Applications of Lipidic Nanocarrier Systems. Pharmaceutics.

[36] Lam H.T., Le-Vinh B., Phan T.N.Q., Bernkop-Schnürch A. (2019). Self-emulsifying drug delivery systems and cationic surfactants: Do they potentiate each other in cytotoxicity?. J. Pharm. Pharmacol..

[37] Gonzalez-Paredes A., Torres D., Alonso M.J. (2017). Polyarginine nanocapsules: A versatile nanocarrier with potential in transmucosal drug delivery. Int. J. Pharm..

[38] Duan F., Feng X., Yang X., Sun W., Jin Y., Liu H., Ge K., Li Z., Zhang J. (2017). A simple and powerful co-delivery system based on pH-responsive metal-organic frameworks for enhanced cancer immunotherapy. Biomaterials.

[39] Wusiman A., Xu S., Ni H., Gu P., Liu Z., Zhang Y., Qiu T., Hu Y., Liu J., Wu Y. (2019). Immunomodulatory effects of Alhagi honey polysaccharides encapsulated into PLGA nanoparticles. Carbohydr. Polym..

[40] Bass J.J., Wilkinson D.J., Rankin D., Phillips B.E., Szewczyk N.J., Smith K., Atherton P.J. (2017). An overview of technical considerations for Western blotting applications to physiological research. Scand. J. Med. Sci. Sports.

[41] Lin J., Zhang H., Chen Z., Zheng Y. (2010). Penetration of Lipid Membranes by Gold Nanoparticles: Insights into Cellular Uptake, Cytotoxicity, and Their Relationship. ACS Nano.

[42] Ganguly P., Breen A., Pillai S.C. (2018). Toxicity of Nanomaterials: Exposure, Pathways, Assessment, and Recent Advances. ACS Biomater. Sci. Eng..

[43] Li J.C., Mao H.L., Kawazoe N., Chen G.P. (2017). Insight into the interactions between nanoparticles and cells. Biomater. Sci..

[44] Giang J., Seelen M.A.J., van Doorn M.B.A., Rissmann R., Prens E.P., Damman J. (2018). Complement Activation in Inflammatory Skin Diseases. Front. Immunol..

[45] Ghebrehiwet B. (2016). The complement system: An evolution in progress. F1000Research.

[46] Liu Y., Yin Y., Wang L., Zhang W., Chen X., Yang X., Xu J., Ma G. (2013). Engineering Biomaterial-Associated Complement Activation to Improve Vaccine Efficacy. Biomacromolecules.

[47] Peleteiro M., Presas E., González-Aramundiz J.V., Sánchez-Correa B., Simón-Vázquez R., Csaba N., Alonso M.J., González-Fernández Á. (2018). Polymeric Nanocapsules for Vaccine Delivery: Influence of the Polymeric Shell on the Interaction With the Immune System. Front. Immunol..

[48] HogenEsch H., O’Hagan D.T., Fox C.B. (2018). Optimizing the utilization of aluminum adjuvants in vaccines: You might just get what you want. npj Vaccines.

[49] Alvarez-Figueroa M.J., González-Aramundiz J.V. (2008). Passive and Iontophoretic Transdermal Penetration of Chlorpromazine. Pharm. Dev. Technol..

[50] Jung E.C., Maibach H.I. (2015). Animal models for percutaneous absorption. J. Appl. Toxicol..

[51] Alvarez-Figueroa M.J., Abarca-Riquelme J.M., González-Aramundiz J.V. (2019). Influence of protamine shell on nanoemulsions as a carrier for cyclosporine-A skin delivery. Pharm. Dev. Technol..

[52] Bos J.D., Meinardi M.M.H.M. (2000). The 500 Dalton rule for the skin penetration of chemical compounds and drugs. Exp. Dermatol..

[53] Brown M.B., Jones S.A. (2005). Hyaluronic acid: A unique topical vehicle for the localized delivery of drugs to the skin. J. Eur. Acad. Dermatol. Venereol..

[54] Yang J.-A., Kim E.-S., Kwon J.H., Kim H., Shin J.H., Yun S.H., Choi K.Y., Hahn S.K. (2012). Transdermal delivery of hyaluronic acid—Human growth hormone conjugate. Biomaterials.

[55] Kim H., Jeong H., Han S., Beack S., Hwang B.W., Shin M., Oh S.S., Hahn S.K. (2017). Hyaluronate and its derivatives for customized biomedical applications. Biomaterials.

[56] Jung H.S., Kim K.S., Yun S.H., Hahn S.K. (2014). Enhancing the transdermal penetration of nanoconstructs: Could hyaluronic acid be the key?. Nanomedicine.

